# Coats' Disease Diagnosed During Adulthood

**DOI:** 10.7759/cureus.16303

**Published:** 2021-07-10

**Authors:** Rahaf A Mandura, Abdullah S Alqahtani

**Affiliations:** 1 Department of Ophthalmology, King Abdulaziz University, Jeddah, SAU; 2 Department of Ophthalmology, King Saud Bin Abdulaziz University for Health Sciences, King Abdulaziz Medical City, Jeddah, SAU

**Keywords:** coat's disease, adult-onset, macroaneurysm, telangiectasia, visual loss

## Abstract

Coats' disease is an idiopathic non-hereditary condition first described by Coats in 1908 as a congenital retinal telangiectatic and aneurysmal disease associated with retinal exudation. Its presentation is classically in early childhood. We report a rare case of Coats' disease that first presented during adulthood in a 35-year-old male. The patient presented with visual loss in the left eye for two months. His visual acuity was counting fingers in the left eye and fundus examination revealed extensive lipid exudation in the macula with telangiectatic vessels and microaneurysms with vascular malformation in the inferotemporal quadrant. Fluorescein angiography showed leakage from the telangiectatic vessels, and optic coherence tomography showed significant macular edema. A provisional diagnosis of adult-onset Coats’ disease was made. The patient responded well to intravitreal ranibizumab injections for macular edema and sectoral argon laser photocoagulation for peripheral vascular abnormalities. This case is unusual in adulthood onset and the first presentation was during adulthood in the third decade of life in contrast to the typical age of onset which is younger than five years.

## Introduction

Coats' disease, also known as retinal telangiectasia, is an idiopathic non-hereditary condition. It was first described by Coats in 1908 as a congenital retinal telangiectatic and aneurysmal disease associated with retinal exudation that may progress to massive exudative retinal detachment [1]. The classical presentation of the disease is usually in early childhood under the age of five years. However, it has been reported as a rare entity occurring in adults aged more than 35 years and referred to as 'adult-onset Coats' disease' [2-6]. Similar to the typical childhood presentation, it can be unilateral with male predilection accompanied by vascular telangiectasis, lipid exudation, and macular edema. However, it is less extensive with slower progression and better response to treatment than that of children [4]. In children, it can lead to vision loss, strabismus or leukocoria while in adults, the visual acuity is usually not significantly affected unless exudation involves the macula, which requires treatment. Regarding treatment modalities, laser photocoagulation, anti-vascular endothelial growth factor (anti‐VEGF) injections, and cryotherapy are considered effective treatments for Coats' disease [2]. We report a case of Coats' disease which is unusual in that the age of onset and the first presentation was during adulthood in the third decade of life in contrast to the typical age of onset that is younger than five years of age.

## Case presentation

**Figure 1:**
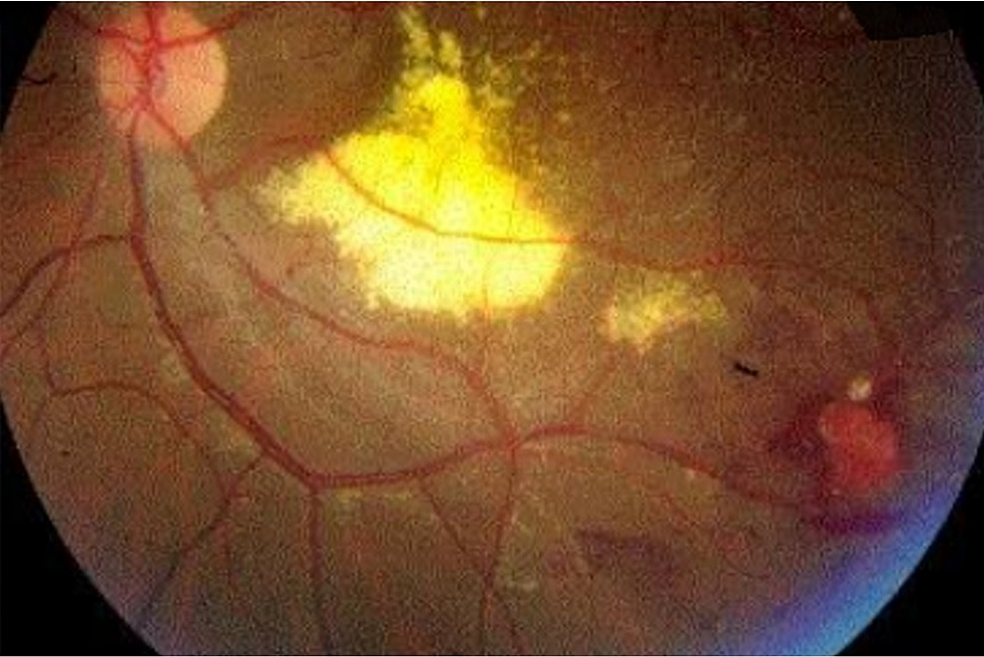
Fundus photo of the left eye showing lipid exudates in macular area and peripheral inferotemporal vasculopathy with adjacent retinal hemorrhage

A 35-year-old male patient presented to the outpatient clinic complaining of decreased vision in his left eye for a duration of two months. There was no associated pain, photophobia, or redness, no history of trauma, no history suggestive of any previous episode of ocular inflammation, and no previous laser treatment. The patient had no prior history of any systemic diseases such as diabetes, hypertension, and hypercholesterolemia. On eye examination, his best-corrected visual acuity (BCVA) at presentation in the right eye (OD) was 20/20 while the left eye (OS) was limited to counting fingers at a distance of 50 cm. In addition, anterior segments were normal in both eyes (OU). Fundus examination (OD) was unremarkable, and (OS) showed extensive lipid exudates in the macular area involving the fovea, forming a plaque-like exudate that measured around two-disc diameter vertically, two and a half-disc diameter horizontally with areas of microaneurysms and telangiectatic vessels. Peripheral inferotemporal vascular malformation with adjacent retinal hemorrhage was also seen (Figure 1).

**Figure 2:**
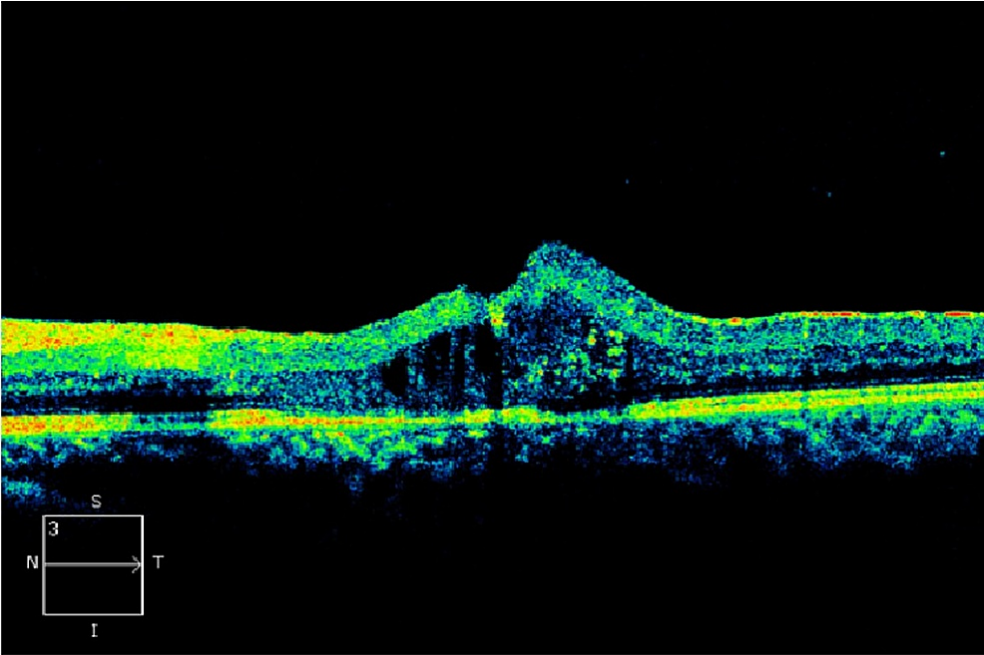
Optical coherence tomography of the left eye showing macular edema

However, there were no signs of inflammation or vasculitis, drusen, retinal pigment epithelial changes, or evidence of healed choroiditis in both eyes. Optical coherence tomography (OCT) (OS) showed significant macular edema, while (OD) was unremarkable (Figure 2).

**Figure 3:**
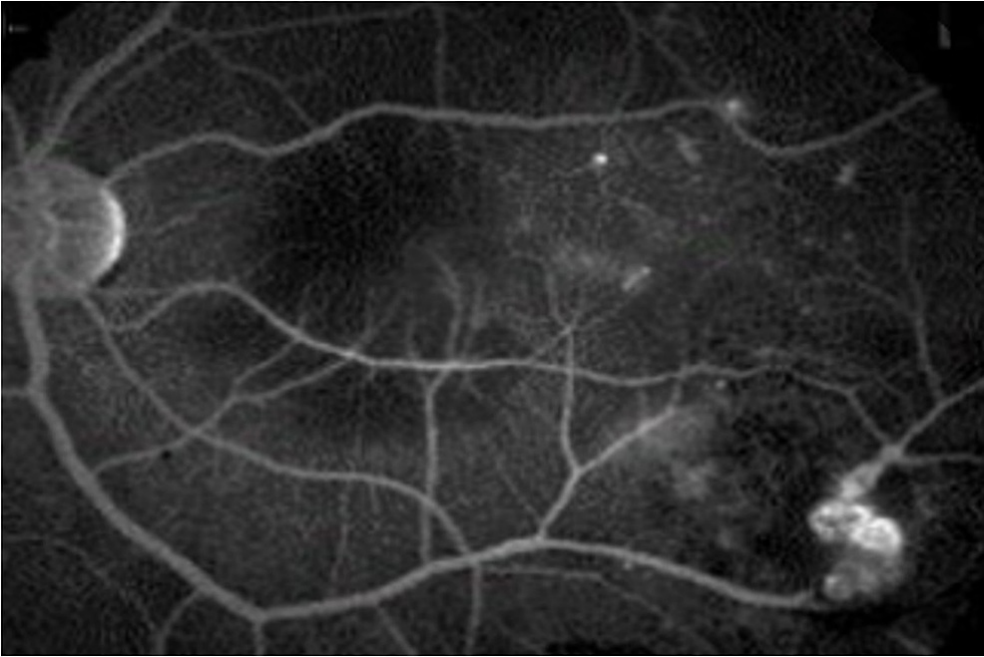
Fundus fluorescein angiography of the left eye showing leakage from the telangiectatic vessels in the macula and the inferotemporal quadrant

Fundus fluorescein angiography (FFA) (OS) showed a hypofluorescent area in the macula due to blockage of the background fluorescence by macular lipid exudates and leakage from telangiectatic vessels. There was also a well-defined inferotemporal hyperfluorescent area corresponding to abnormal vasculature with leakage, and FFA (OD) was normal (Figure 3). Based on clinical presentation, the patient’s provisional diagnosis was adult-onset Coats' disease. Following informed consent, treatment was started in the form of intravitreal anti-vascular endothelial growth factor therapy (anti-VGEF) ranibizumab (Novartis' Lucentis 0.05 ml containing 0.5 mg) injection (OS) for macular edema followed by focal laser photocoagulation for the peripheral vascular malformation inferotemporally. Four weeks later, the patient showed good response, and his examination showed BCVA that improved to 20/400 (OS), and the fundus exam showed improvement with residual scattered lipid exudates in the macula. Furthermore, OCT showed some resolution of macular edema.

As a result, intravitreal ranibizumab was repeated in the left eye. The second follow up four weeks later, his BCVA improved to 20/60, and OCT showed complete resolution of macular edema. The patient received four more ranibizumab injections at a monthly interval, but no further response was seen on the subsequent visits. Long term follow-up after three years showed that his BCVA remained stable at 20/60 (OS), fundus examination showed scattered lipid exudates, and OCT showed clear macula.

## Discussion

Coats' disease is an idiopathic, unilateral exudative retinal vascular abnormality manifested as telangiectasia and aneurysmal vascular dilations. It has variable severity and can range from isolated vascular abnormalities without lipid exudation to massive intra- and subretinal lipid exudates deposition [2]. Coats' is classically reported among young male patients and the disease is diagnosed in adulthood as a rare condition [2-6] and has been described in the literature in rare case reports with onset at 56 years [3] and 47 years [7], and case series with a presentation between 22 and 62 years of age [8].

Adult-onset Coats’ disease is characterized by retinal vascular abnormalities which are similar to those seen in younger patients. In the pediatric group, the disease is usually progressive occurring in boys with a peak incidence between 6-8 years of age, while the adult form usually presents over the age of 35 years [3]. In mild cases, one or more localized foci of retinal telangiectasia are noted within the retinal capillary bed, typically in the temporal quadrants between the equator and Ora Serrata [6]. Adult-onset Coats' disease phenotypically differs from its counterpart in children in that it is less symptomatic, may have good visual acuity, limited area of retinal involvement, and slower progression of the disease. Meanwhile, the vascular anomalies generally appear more peripheral and localized, and retinal hemorrhages are more common because of bleeding from ruptured aneurysms [5]. The staging system proposed by Shields et al. in 2002 defined the progression to exudative retinal detachment as stage 3 disease [9]. Rishi et al. published a series of adult-onset Coats’, which showed that 10 (21%) of their 48 adult-onset cases had exudative, stage 3, retinal detachments [10]. This is by far less than the 81% of cases found with exudative retinal detachment in a large case series study on the childhood onset of Coats’ by Shields et al. [9]. Using the same staging system, our adult-onset case, having foveal macular exudates, microaneurysms, and telangiectasia is classified as stage 2B disease.

In line with the published case series by Bottini et al., they described two cases of adult-onset Coats’ disease with predominant arterial macroaneurysms with hemorrhages and exudation and minimal telangiectatic changes [11]. In comparison, our case showed similar findings with minimal telangiectasia that otherwise is considered a predominant feature in childhood-onset Coats' disease. Although systemic medical conditions such as hypertension, diabetes mellitus, and hypercholesterolemia have been reported as an association with adult-onset Coats disease [12]. However, our patient has no such association with no prior medical history of any systemic diseases. The aim of the management of adult-onset Coats' is to occlude telangiectatic vessels and to promote the reabsorption of lipid exudates, which results in improvement of visual acuity. Similar to what Dhawan et al. reported in their study, our case had macular lipid exudates with macular edema and inferotemporal vascular malformation, which improved with intravitreal ranibizumab injection and sectoral argon laser photocoagulation, respectively [3].

## Conclusions

Coats' disease is primarily a disease of childhood though we should consider its diagnosis in adult patients presenting with extensive retinal lipid exudation and prominent vasculopathy. The natural course of adult-onset Coats' disease is better than that of children. However, treatments should not be delayed if the disease is macular threatening to halt its progression and further deterioration of vision.
